# Supplementary material for: Opposite microglial activation stages upon loss of PGRN or TREM2 result in reduced cerebral glucose metabolism
Source: EMBO Mol Med. 2019 May 23;11(6):e9711. doi: 10.15252/emmm.201809711 (PMC6554672; doi:10.15252/emmm.201809711)
Supplement: Supplementary file 1 — Expanded View Figures PDF [file EMMM-11-e9711-s001.pdf]

Expanded View Figures

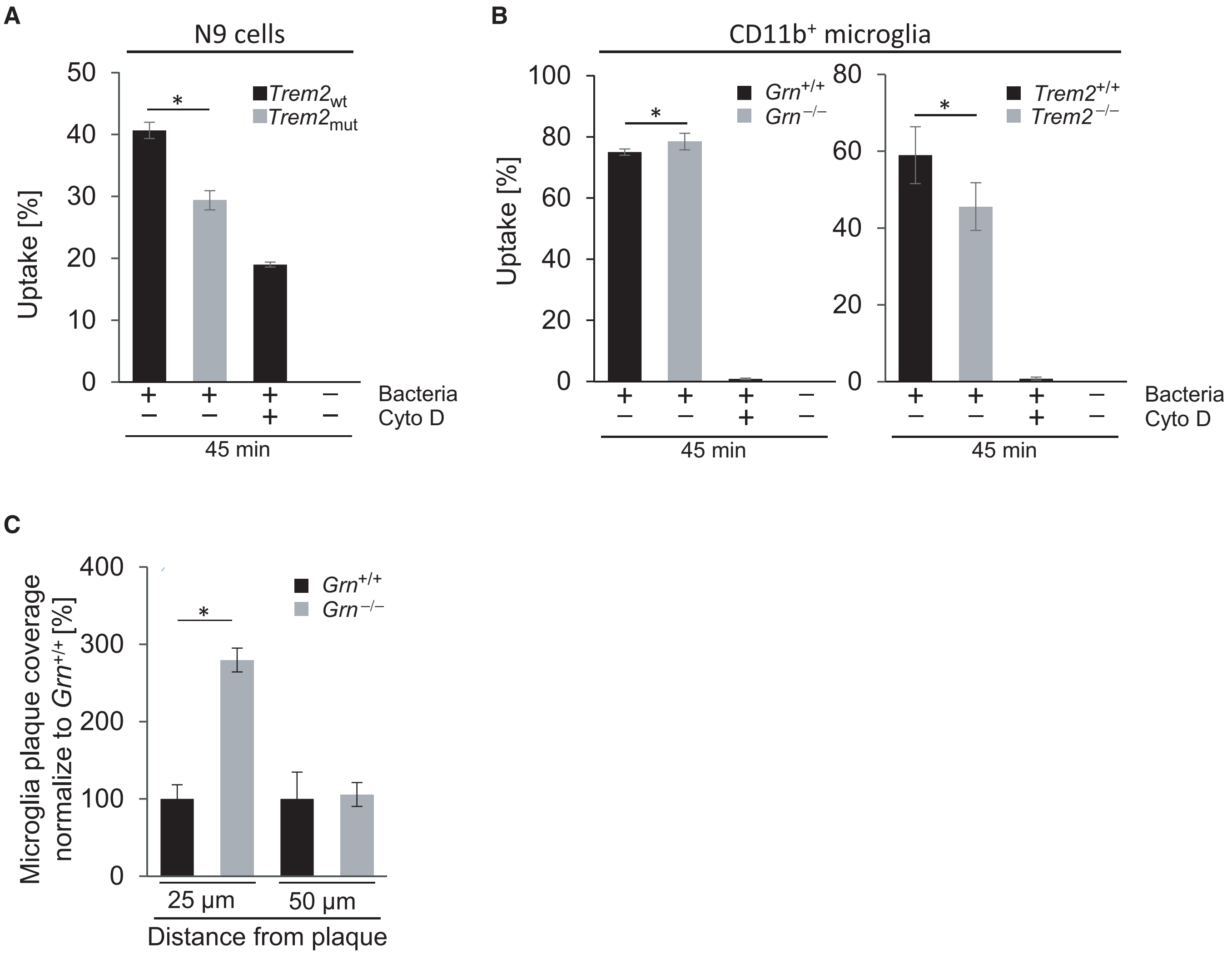

Figure EV1. Reduced phagocytosis upon TREM2 deficiency in contrast to enhanced phagocytosis and clustering around amyloid plaques in the absence of PGRN.

A Flow cytometric analysis of phagocytic capacity in N9 $Trem2_{wt}$ and N9 $Trem2_{mut}$ cells using pHrodo green *E. coli* as target particles. Phagocytosis was terminated after 45 min of incubation. Data are presented as mean percentage of cells positive for pHrodo uptake ± SD (n = 3).

B Flow cytometric analysis of phagocytic capacity of cultured primary microglia from wild-type, $Grn^{-/-}$, and $Trem2^{-/-}$ mice (2 months of age, male) using pHrodo green *E. coli* as target particles. Phagocytosis was terminated after 45 min of incubation. Left panel: Data are presented as mean percentage of $CD11b^{+}$ cells positive for pHrodo uptake ± SD (three biological replicates). Right panel: Data are presented as mean percentage of $CD11b^{+}$ cells positive for pHrodo uptake ± SD (two biological replicates).

C Percentage IBA1-positive microglia within given plaque distance quantified from immunohistochemical stainings shown in Fig 3E. Data are normalized to APPPS1/ $Grn^{+/+}$ mice (n = 4; 4-month-old; two males and two females per genotype). Data are shown as mean ± SD.

Data information: For statistical analysis, unpaired two-tailed Student's t-test was performed (A, B) between TREM2 or PGRN-deficient microglia against wild-type microglia, and the Mann–Whitney U-test, two-tailed analysis (C) was performed between genotypes. Significance is indicated by $^{*}P < 0.05$.

Source data are available online for this figure.

© 2019 The Authors
