# Supplementary material for: Opposite microglial activation stages upon loss of PGRN or TREM2 result in reduced cerebral glucose metabolism
Source: EMBO Mol Med. 2019 May 23;11(6):e9711. doi: 10.15252/emmm.201809711 (PMC6554672; doi:10.15252/emmm.201809711)
Supplement: Supplementary file 3 — Source Data for Expanded View [file EMMM-11-e9711-s005.pdf]

**Fig EV1A**	N9 cell			
time	45 min		Cyto D	w/o E.coli
cell line	**Trem2 wt**	**Trem2 mut**	**Trem2 wt**	**Trem2 wt**
n	3	3	3	3
Mean uptake [%]	40.68	29.36	18.99	0
SD	1.32	0.40	1.55	0.00
(T-Test) P-value	0.02923			

**Fig EV1B**	Microglia				Microglia			
time	45 min		Cyto D	w/o E.coli	45 min		Cyto D	w/o E.coli
cell line	**Grn+/+**	**Grn-/-**	**Grn+/+**	**Grn+/+**	**Trem2+/+**	**Trem2-/-**	**Trem2+/+**	**Trem2+/+**
biological replicates (n)	3	3	3	3	2	2	2	2
Mean uptake [%]	75.02	78.46	0.85	0.00	58.98	45.61	0.81	0.00
SD	1.0	2.7	0.3	0.0	7.4	6.2	0.4	0.0
(T-Test) P-value	0.046				0.007			

**Fig EV1C**	**25 µm**		**50 µm**	
genotype	**APPPS/Grn$^{+/+}$**	**APPPS/Grn$^{-/-}$**	**APPPS/Grn$^{+/+}$**	**APPPS/Grn$^{-/-}$**
n	4	4	4	4
microglia plaque coverage	115.8	296.6	116.8	127.8
in % of APPPS/Grn+/+	115.8	274.9	140.8	101.5
	84.6	260.7	70.0	101.4
	83.8	286.5	72.3	92.1
normalized microglia/plaque	**100.0**	**279.6**	**100.0**	**105.7**
SD %	18.25	15.45	34.71	15.38
(Mann Whitney test) P-value		0.0286		0.8857

Source Data Figure EV1
Götzl, Brendel, Werner et al. 2019
