# Supplementary material for: Opposite microglial activation stages upon loss of PGRN or TREM2 result in reduced cerebral glucose metabolism
Source: EMBO Mol Med. 2019 May 23;11(6):e9711. doi: 10.15252/emmm.201809711 (PMC6554672; doi:10.15252/emmm.201809711)
Supplement: Supplementary file 5 — Source Data for Figure 2 [file EMMM-11-e9711-s003.zip › emmm201809711-sup-0003-SDataFig2A.pdf]

Fig 2B	CD11^{+} microglia											
protein	ApoE		CLEC7A		TREM2$_{im}$		TREM2$_{m}$		CD68		IBA1	
cell line	**Grn$^{+/+}$**	**Grn$^{-/-}$**	**Grn$^{+/+}$**	**Grn$^{-/-}$**	**Grn$^{+/+}$**	**Grn$^{-/-}$**	**Grn$^{+/+}$**	**Grn$^{-/-}$**	**Grn$^{+/+}$**	**Grn$^{-/-}$**	**Grn$^{+/+}$**	**Grn$^{-/-}$**
A	1.62	2.57	1.36	4.49	0.82	2.48	0.94	0.61	1.44	4.26	0.99	0.94
B	0.79	3.26	0.89	2.47	1.48	1.28	1.04	0.51	1.25	6.32	0.96	0.85
C	0.59	3.17	0.75	2.29	0.70	1.41	1.02	0.35	0.31	8.73	1.06	1.03
n	3	3	3	3	3	3	3	3	3	3	3	3
Mean	1.00	3.00	1.00	3.08	1.00	1.72	1.00	2.69	1.00	6.44	1.00	0.94
SD	0.42	0.29	0.24	0.94	0.32	0.50	0.20	0.21	0.46	1.53	0.04	0.06
(T-Test) P-value	0.00648		0.04618		0.18542		0.00168		0.01528		0.35426	

Fig 2C	sTREM2 - brain							
age group	**3-4 m**		**6 m**		**13 m**		**16-18 m**	
genotype	**Grn$^{+/+}$**	**Grn$^{-/-}$**	**Grn$^{+/+}$**	**Grn$^{-/-}$**	**Grn$^{+/+}$**	**Grn$^{-/-}$**	**Grn$^{+/+}$**	**Grn$^{-/-}$**
female	**1**	**1**	**5**	**6**	**4**	**2**	**3**	**3**
male	**3**	**2**			**2**	**2**	**2**	**2**
n	4	3	5	6	6	4	5	5
Mean pg/mg	163.91	217.01	168.04	204.70	161.50	225.86	186.07	249.28
SD	6.25	27.63	21.10	17.70	14.89	22.17	30.97	43.87
SEM	3.13	15.95	9.43	7.23	6.08	11.08	13.85	19.62
(T-Test) P-value		0.01221		0.01194		0.00054		0.03008

Fig 2D	sTREM2 serum							
age group	**3 m**		**6 m**		**13 m**		**16-18 m**	
genotype	**Grn$^{+/+}$**	**Grn$^{-/-}$**	**Grn$^{+/+}$**	**Grn$^{-/-}$**	**Grn$^{+/+}$**	**Grn$^{-/-}$**	**Grn$^{+/+}$**	**Grn$^{-/-}$**
female	**3**	**4**	**10**	**5**	**8**	**1**	**1**	**1**
male	**4**	**2**	**3**	**4**	**2**	**3**	**5**	**5**
n	7	6	13	9	10	4	6	6
Mean ng/ml	10.10	17.27	10.45	23.48	12.44	29.50	14.15	28.97
SD	3.51	4.84	4.36	6.93	2.71	8.57	2.18	6.81
SEM	1.33	1.97	1.21	2.31	0.86	4.28	0.89	2.78
(T-Test) P-value		0.01025		0.00003		0.00007		0.00048

Fig 2F	Cortex								
protein	**wild type**			**Grn$^{-/-}$**			**Trem2$^{-/-}$**		
cell line	IBA1	**P2RY12**	**% P2RY12 / IBA1**	IBA1	**P2RY12**	**% P2RY12 / IBA1**	IBA1	**P2RY12**	**% P2RY12 / IBA1**
A	7.74	7.94	102.51	6.75	4.44	65.78	4.53	6.48	143.01
B	5.69	4.61	81.07	5.92	3.64	61.45	3.95	6.04	152.87
C	4.27	4.04	94.59	7.98	4.53	56.79	2.47	5.92	240.08
n	3	3		3	3		3	3	
Mean			92.72			61.34			178.66
SD			10.84			4.50			53.42
Tukey's multiple comparisons test		**adj. p-value**	**significance**						
	wt vs. Grn$^{-/-}$	*0.5423*	*ns*						
	wt vs. Grn$^{-/-}$	*0.0489*	*						
	Grn$^{-/-}$ vs. Trem2$^{-/-}$	*0.0135*	*						

Fig 2H	BV2										BV2			
protein	ApoE_medium		ApoE_ lysat		CLEC7A_ lysat		P2RY12_ lysat		TREM2 Lysat		APPs_medium		Calnexin Lysat	
cell line	Grn wt	Grn mut	Grn wt	Grn mut	Grn wt	Grn mut	Grn wt	Grn mut	Grn wt	Grn mut	Grn wt	Grn mut	Grn wt	Grn mut
A	1.23	2.46	0.91	3.59	0.78	2.49	0.94	0.61	0.86	1.57	115.87	149.30	92.24	143.57
B	0.86	3.30	0.97	5.04	1.04	2.87	1.04	0.51	1.06	2.05	91.64	97.67	88.51	148.42
C	0.91	4.07	1.12	5.76	1.18	3.67	1.02	0.35	0.85	1.54	92.50	84.82	85.92	114.41
D									1.18	1.69			113.31	86.89
E									1.06	1.71			120.02	78.76
n	3	3	3	3	3	3	3	3	5	5	3	3	5	5
Mean	1.00	3.28	1.00	4.80	1.00	3.01	1.00	0.49	1.00	1.71	100.00	110.60	100.00	114
SD	0.20	0.81	0.11	1.11	0.21	0.61	0.06	0.13	0.14	0.21	13.75	34.13	16	32
(T-Test) P-value	0.00903		0.00407		0.00555		0.00370		0.00021		0.64406		0.3889	

Fig 2I	BV2	
protein	sTREM2_medium	
cell line	Grn wt	Grn mut
A	1.01	1.21
B	1.01	1.06
C	0.97	1.08
D	1.00	1.08
E		
n	4	4
Mean	1.00	1.11
SD	0.014	0.055
(T-Test) P-value	0.02612	

Fig 2J	BV2 mRNA							
gene	***Apoe***		***Clec7a***		***P2ry12***		***Trem2***	
cell line	Grn wt	Grn mut	Grn wt	Grn mut	Grn wt	Grn mut	Grn wt	Grn mut
n	3	3	3	3	3	3	3	3
Mean	1.00	1.57	1.00	1.94	1.00	0.66	1.00	1.50
SD	0.02	0.04	0.09	0.08	0.07	0.03	0.04	0.08
(T-Test) P-value		0.00002		0.00021		0.00163		0.00077

Source Data Figure 2
Götzl, Brendel, Werner et al. 2019

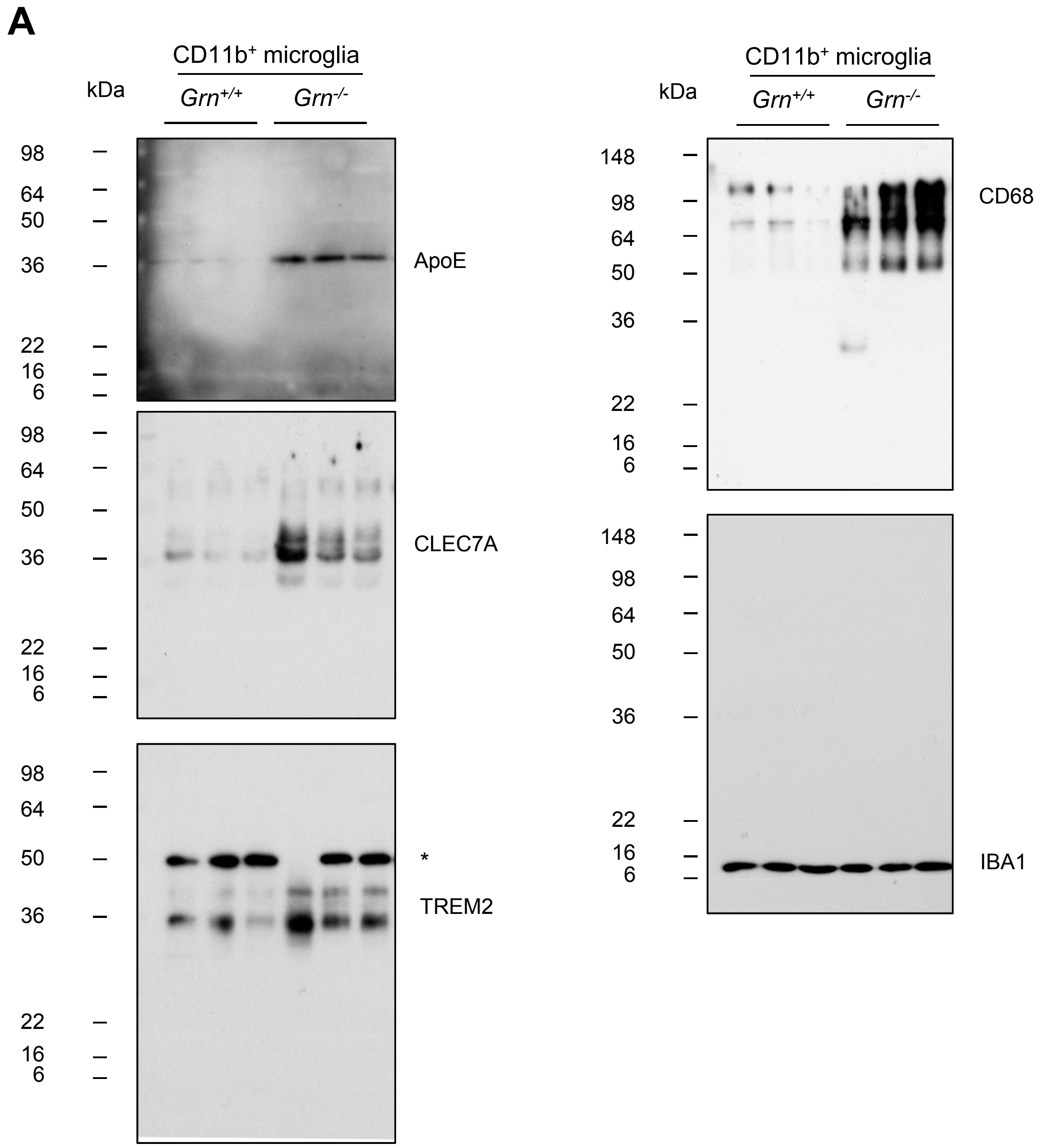

Source Data Figure 2A
Götzl, Brendel, Werner et al. 2019

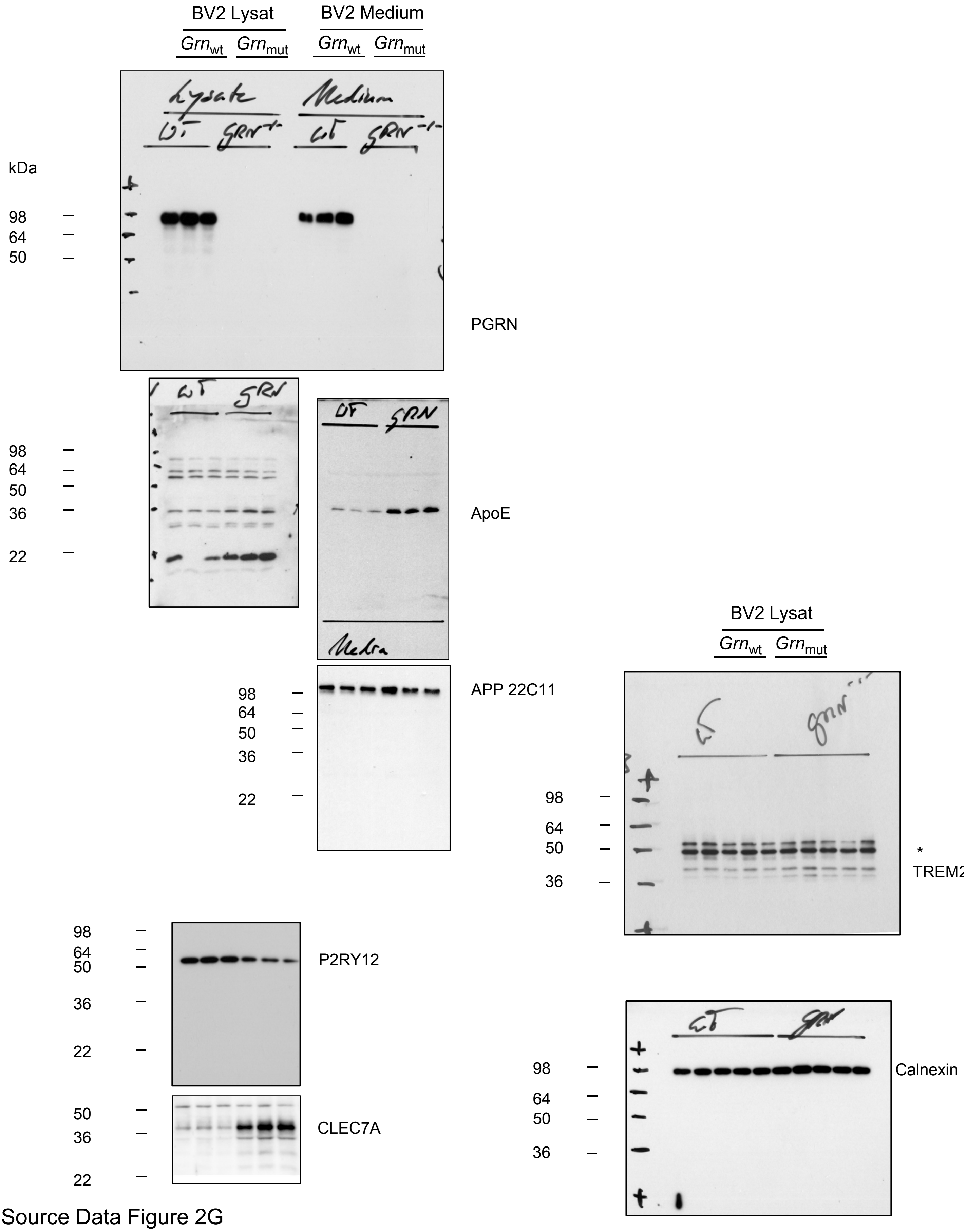

Source Data Figure 2G
Götzl, Brendel, Werner et al. 2019
