# Supplementary figures and images for: Opposite microglial activation stages upon loss of PGRN or TREM2 result in reduced cerebral glucose metabolism
Source: EMBO Mol Med. 2019 May 23;11(6):e9711. doi: 10.15252/emmm.201809711 (PMC6554672; doi:10.15252/emmm.201809711)

**A**

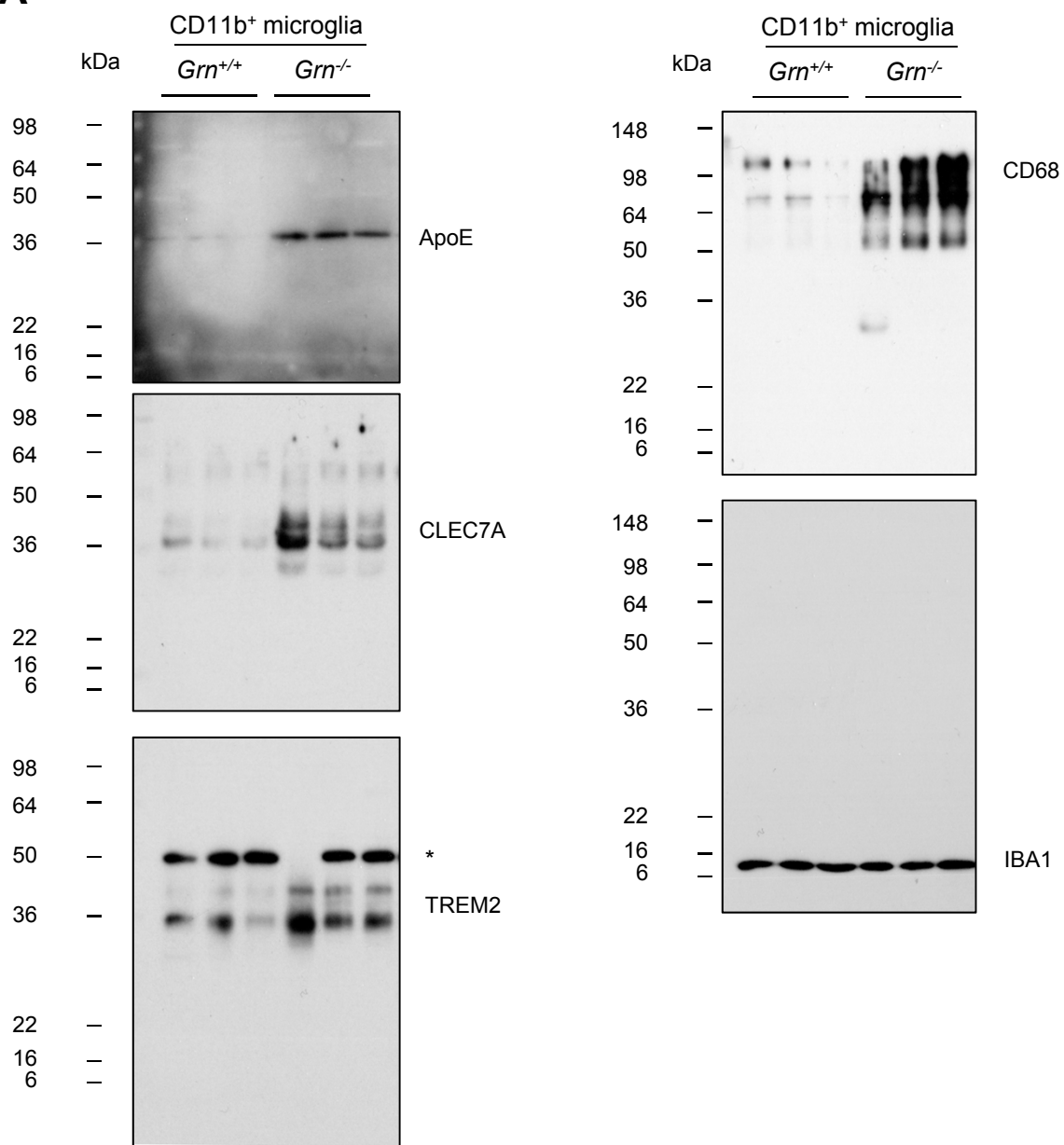

Figure 2A Source Data

Supplement: Supplementary file 5 — Source Data for Figure 2 [file EMMM-11-e9711-s003.zip › emmm201809711-sup-0004-SDataFig2A_WB.pdf]

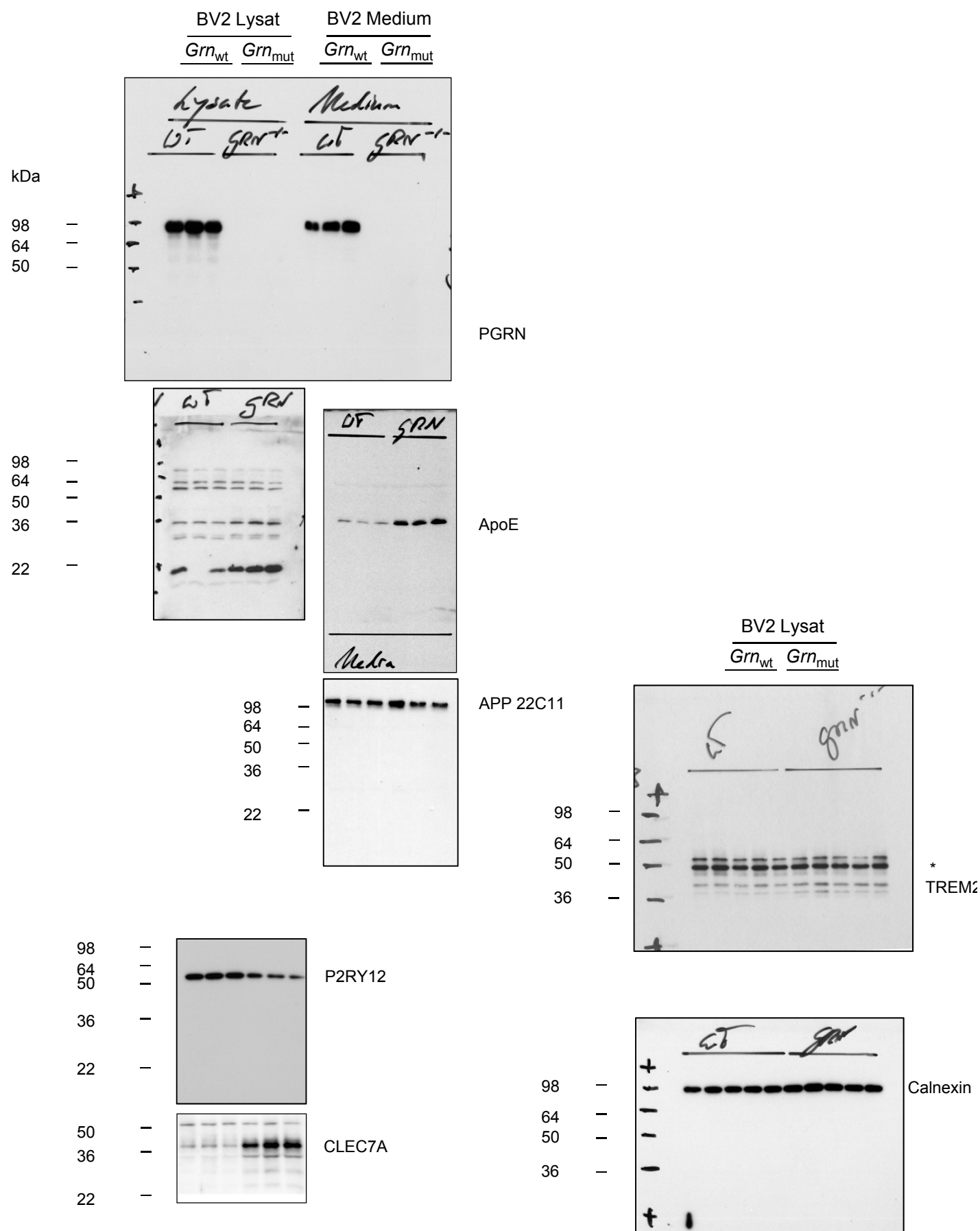

Fig. 2G Source Data

Supplement: Supplementary file 5 — Source Data for Figure 2 [file EMMM-11-e9711-s003.zip › emmm201809711-sup-0005-SDataFig2G_WB.pdf]
