# Supplementary material for: Opposite microglial activation stages upon loss of PGRN or TREM2 result in reduced cerebral glucose metabolism
Source: EMBO Mol Med. 2019 May 23;11(6):e9711. doi: 10.15252/emmm.201809711 (PMC6554672; doi:10.15252/emmm.201809711)
Supplement: Supplementary file 6 — Source Data for Figure 3 [file EMMM-11-e9711-s004.pdf]

**Fig 3A**					
time	30 min		60 min		Cyto D
cell line	**Grn wt**	**Grn mut**	**Grn wt**	**Grn mut**	**Grn wt**
n	3	3	3	3	3
Mean uptake [%]	19.37	29.46	29.33	40.26	12.09
SD	1.76	1.93	1.08	2.51	0.41
(T-Test) P-value	0.00259		0.00229		

**Fig 3C**		
genotype	**$Grn^{+/+}$**	**$Grn^{-/-}$**
n	3	3
Mean distance [µm]	266.52	449.5
SD	28.59	15.04
T-test: unpaired, two tails; P-value		0.00060

**Fig 3D**		
genotype	**$Grn^{+/+}$**	**$Grn^{-/-}$**
n	3	3
number migrated microglia	87.85	417.00
SD	9.89	99.98
T-test: unpaired, two tails; P-value		0.00476

**Fig 3F**		
genotype	**$APPPS/Grn^{+/+}$**	**$APPPS/Grn^{-/-}$**
n	4	4
normalized microglia/plaque	1.000	1.548
SD	0.231	0.171
(Mann-Whitney U test) P-value		0.0286

Source Data Figure 3
Götzl, Brendel, Werner et al. 2019
